# Cereal Coffee as a Functional Additive in Wheat Bread: Impact on Dough and Bread Properties

**DOI:** 10.3390/foods13243991

**Published:** 2024-12-10

**Authors:** Grażyna Cacak-Pietrzak, Justyna Grabarczyk, Anna Szafrańska, Anna Krajewska, Dariusz Dziki

**Affiliations:** 1 Department of Food Technology and Assessment, Institute of Food Sciences, Warsaw University of Life Sciences (WULS), 159C Nowoursynowska Street, 02-776 Warsaw, Poland; grazyna_cacak_pietrzak@sggw.edu.pl; 2 Department of Grain Processing and Bakery, Prof. Wacław Dąbrowski Institute of Agricultural and Food Biotechnology, State Research Institute, 36 Rakowiecka Street, 02-532 Warsaw, Poland; justyna.grabarczyk@ibprs.pl (J.G.); anna.szafranska@ibprs.pl (A.S.); 3 Department of Thermal Technology and Food Process Engineering, University of Life Sciences in Lublin, 31 Głęboka Street, 20-612 Lublin, Poland; anna.krajewska@up.lublin.pl

**Keywords:** baking, water absorption, dough physical properties, antioxidant properties, texture, color, sensory analysis

## Abstract

The chemical composition and quality attributes of wheat bread enriched with cereal coffee were analyzed, with additive incorporated as a partial replacement for wheat flour at levels of 2%, 4%, 6%, 8%, and 10%. The rheological properties of the bread dough, consisting of wheat flour and cereal coffee blends, were evaluated using farinograph and extensograph analyses. Results indicated that the addition of cereal coffee decreased flour water absorption, extended dough stability, and increased dough softening. Dough containing cereal coffee showed greater resistance to stretching and reduced extensibility. However, the incorporation of cereal coffee led to a reduction in bread volume and an increase in crumb hardness and density, especially when the substitution level exceeded 6%. In terms of nutritional composition, the levels of dietary fiber, ash, fat, and total polyphenols increased with higher cereal coffee content, while crumb brightness decreased, and yellowness and redness intensified. Overall, the study suggests that cereal coffee can function as a valuable ingredient in bread; however, substitution levels should ideally be kept below 8% to preserve acceptable sensory qualities.

## 1. Introduction

The increasing awareness among consumers regarding the impact of nutrition on human health is driving producers to incorporate ingredients with health-promoting properties into food formulations. Bread serves as an ideal matrix for enrichment with additional ingredients, given its status as one of the most widely consumed food products worldwide [1]. In Europe, wheat bread is especially popular; however, it is typically made from refined flours high in digestible carbohydrates but low in dietary fiber and bioactive compounds [2]. The health benefits associated with regular consumption of fiber-enriched products are well-documented in the literature. Adequate fiber intake helps lower blood cholesterol levels, thereby reducing the risk of cardiovascular disease [3]. Fiber is also a valuable source of various phenolic compounds with strong antioxidant activity [4]. It slows the digestion of carbohydrates and lowers blood glucose levels, thereby reducing insulin secretion and the risk of diabetes [5]. Fiber consumption increases satiety, helping to limit food intake in subsequent meals and lowering the risk of obesity [6]. Additionally, it has a prebiotic effect, stimulating the growth of beneficial gut bacteria [7,8]. Replacing part of the wheat flour in bread formulations with high-fiber additives can thus increase the dietary intake of this essential component, offering health benefits to consumers. Conversely, the incorporation of high-fiber raw materials into wheat bread affects the dough matrix, primarily composed of gluten, starch, fat, and water. This modification can result in a decline in the dough’s physical properties [9,10] and, subsequently, in the overall quality of the bread [11]. High-fiber bread typically exhibits reduced loaf volume and a denser, less porous crumb structure compared to conventional wheat bread [12]. Additionally, alterations in flavor and aroma [13] pose a challenge in identifying optimal types and quantities of additives that enhance the bread’s nutritional profile without diminishing its sensory qualities and consumer acceptability.

To increase the dietary fiber content in bread, producers may enrich formulations with fibers from various sources [14]. Cereal coffee (CC) is a popular substitute for coffee in Poland and presents an intriguing option as a bread ingredient. This product is derived from roasted and ground barley and rye grains (60–70%), with additions of chicory (15–20%) and sugar beet (6–10%) [15]. During the roasting process, several chemical reactions, such as caramelization and Maillard reactions, occur, leading to protein denaturation, starch gelatinization, and browning reactions that enhance the flavor, color, and aroma of the coffee [16]. Majcher et al. [15] identified 30 volatile aromatic compounds in this product. CC is a rich source of water-soluble dietary fiber from barley, and with the addition of chicory, it also contains inulin [17]. Additionally, it is rich in minerals (such as magnesium, selenium, and calcium), B vitamins, and polyphenols with potent antioxidant properties [16,18,19]. The study conducted by Samsonowicz et al. [19] demonstrated that CC contains the highest amounts of gallic acid (514 mg/kg), followed by coumaric acid (83 mg/kg), 4-hydroxyphenylacetic acid (60 mg/kg), and chlorogenic acid (27 mg/kg). The bioactive components in CC help regulate various physiological processes and positively impact human health [17,18]. CC is a highly nutritious, widely accessible, and relatively affordable product [19,20]. It should also be emphasized that CC is caffeine-free, and thus suitable for children, pregnant and breastfeeding women, and individuals with cardiovascular conditions, such as hypertension [20].

The objective of our research was to evaluate the influence of CC on the physical characteristics of wheat dough, as well as the chemical composition, physical characteristics, and sensory attributes of bread. The literature suggests that CC may serve as a valuable ingredient in wheat-based pasta [20]; however, to our knowledge, it has not yet been used as a bread additive, which prompted our investigation.

## 2. Materials and Methods

### 2.1. Materials

In this study, reagents of analytical purity were used for chemical analyses including sodium salicylate, gallic acid, ferrozine (3-(2-pyridyl)-5,6-bis-(4-phenyl-sulfonic acid)-1,2,4-triazine), and ABTS (2,2′-azino-bis-(3-ethylbenzothiazoline-6-sulfonic acid)) (Sigma-Aldrich, Poznań, Poland).

Bread dough was prepared using type 550 wheat flour (WF) supplied by Polskie Młyny in Warsaw, Poland. This flour was characterized by a wet gluten yield of 25.4% ± 0.1, a gluten index (GI) of 90 ± 1, and a falling number of 339 s ± 3. The additional components used were compressed baker’s yeast sourced from Lesaffre Polska (Wołczyn, Poland), salt obtained from Cenos (Września, Poland), and CC “Kujawianka” provided by Delecta (Włocławek, Poland). According to the manufacturer’s label, CC “Kujawianka” comprised 60% roasted rye, 20% barley, as well as chicory and sugar beet. CC was ground using an analytical mill (model A11, IKA, Staufen, Germany) to achieve a particle size below 1.0 mm.

Prior to analysis, blends of wheat flour and powdered CC blends were prepared, with wheat flour partially replaced by CC at levels of 2%, 4%, 6%, 8%, and 10%. The raw materials were stored in dark, airtight containers in a room with a temperature of 22–24 °C and relative humidity of 60–65%.

### 2.2. Dough Farinographic Analysis

The farinographic properties of the control dough (made exclusively with wheat flour) and mixtures of CC with wheat flour were evaluated with a Brabender Farinograph-E (Duisburg, Germany) in accordance with the guidelines of the AACC 54-21 method. Water absorption was determined based on the amount of distilled water needed to achieve a consistency of 500 FU. From the standard curve generated using Farinograph v.5 software, parameters such as the time required for dough development, its stability, and the degree of softening after 12 min were recorded.

### 2.3. Dough Extensographic Analysis

The extensographic properties of the control dough and the CC–wheat flour mixtures were evaluated with an Extensograph-E (Brabender, Duisburg, Germany) following the procedures outlined in method 54-10 of the AACC guidelines [21]. Key rheological metrics, including dough energy, extensibility, maximum resistance to stretching, and the D coefficient (calculated by dividing maximum resistance by extensibility), were obtained from the extensograph curve. Dough energy and the D coefficient were measured after intervals of 45, 90, and 135 min of fermentation.

### 2.4. Bread Production Methodology

The control dough recipe included 700 g of wheat flour, 21 g of compressed yeast, 10.5 g of salt, and an amount of water adjusted to achieve a consistency of 350 FU, based on farinographic water absorption. In the experimental doughs, part of the wheat flour was replaced with powdered CC at levels of 2%, 4%, 6%, 8%, and 10% of the total flour weight. The doughs were prepared according to the single-stage method described by Cacak-Pietrzak et al. [22]. The ingredients were blended using an SP-800A mixer (Spar Food Machinery, Taichung, Taiwan) at a medium setting (level 2) for 4 min. Fermentation was conducted in a D-32 chamber (Sveba Dahlen, Fristad, Sweden) at 30 °C and 85% ± 3 relative humidity for 90 min, with the dough punched down after 60 min for one minute. Following fermentation, the dough was portioned into 250 g pieces, shaped manually, placed into baking pans, and left to undergo final proofing in a D-32 fermentation chamber (Sveba Dahlen, Fristad, Sweden) at 30 °C and 85% ± 3 relative humidity for 40 min. The dough was baked in a D-32 oven (Sveba Dahlen, Fristad, Sweden) at 230 °C. The baking time was 30 min. Following baking, the loaves were taken out of the molds and left to cool. They were then packaged in polyethylene bags and kept at a temperature of 22–24 °C for 24 h.

### 2.5. Chemical Composition Analysis of Ingredients and the Final Product

The chemical composition of the wheat flour, CC, and the final bread product was analyzed in detail using the methodologies outlined in AACC [21] and AOAC [23]. Moisture content was measured following AACC 44-15.02. Total protein content was measured using AACC 46-11.02, while fat content was determined by Soxhlet extraction (AACC 30.10.01). Ash content was determined through incineration (AACC 08-01.01). Total dietary fiber, including its soluble and insoluble fractions, was determined using a gravimetric–enzymatic method (AACC Method 32-07 and AOAC 991.43). The digestible carbohydrate content was determined by subtracting the total mass of water, ash, protein, fat, and dietary fiber from 100%.

### 2.6. Physical Characteristics of Bread

After 24 h of cooling, the loaves were weighed to calculate baking loss, yield, loaf volume, density, and crumb texture parameters. The loaf volume was assessed using a NextEngine 3D scanner (Los Angeles, CA, USA) and analyzed with MeshLab software (2023.1), developed by the ISTI-CNR Research Center in Rome (Italy). The measured volume was standardized to 100 g of bread. Crumb density was measured by cutting 25 cm^3^ cubes from the center of the crumb and determining their mass to calculate the density. Crumb texture was evaluated with a TA.XT2i (Stable Microsystems, Surrey, UK). Crumb samples, 20 mm thick and 30 mm in diameter, were compressed twice with a 25 mm probe. The probe moved at a speed of 1 mm/s, with the penetration depth set to 40%. A 45 s pause was introduced between the first and second compressions. The resulting curves were analyzed to determine textural properties like hardness, cohesiveness, resilience, and springiness, as detailed in Cacak-Pietrzak et al. [22].

### 2.7. Color Analysis of Raw Materials, Crust, and Crumb

The color characteristics of the wheat flour, CC, and the bread crumb were analyzed using the CIE-Lab* color system, where L* represents lightness, a* indicates red–green balance, and b* denotes yellow–blue balance. Color measurements were performed with a CR-200 Konica Minolta colorimeter (Osaka, Japan). The difference in color (ΔE*) between the crumb of the control bread and the CC-enriched bread was determined based on the formula given [24].

### 2.8. Analysis of Polyphenol Content and Antioxidant Activity in Raw Materials and Bread

The total polyphenol content, along with the capacity to scavenge DPPH and ABTS•+ radicals, was determined in both the wheat flour and CC, as well as in the bread.

#### 2.8.1. Preparation of Extracts for Polyphenol Content and Antioxidant Activity Analysis

Methanolic extracts from the wheat flour, CC, and bread samples were prepared according to the method outlined by Krajewska et al. [25].

#### 2.8.2. Total Polyphenol Content

The total polyphenol content was quantified using the Folin–Ciocalteu method, following the procedure outlined by Michalska et al. [26]. Absorbance at 720 nm was measured using a UV Mini 1240 Shimadzu spectrophotometer (Kyoto, Japan). Results were expressed as mg GAE per gram of dry matter.

#### 2.8.3. DPPH Free Radical Scavenging Activity

DPPH free radical scavenging activity was evaluated spectrophotometrically following the method of Brand-Williams et al. [27]. A buffer extract was combined with ethanol-based DPPH solution and allowed to react for 15 min. Absorbance at 725 nm was recorded using a microplate spectrophotometer (BioTek, Santa Clara, CA, USA). The scavenging capacity was expressed as EC_50_.

#### 2.8.4. ABTS•+ Cation Radical Scavenging Activity

ABTS•+ radical scavenging activity was assessed spectrophotometrically using the method described by Re et al. [28]. A 0.04 mL buffer extract was mixed with 1.8 mL of the ABTS•+ solution, and absorbance was recorded after 5 min at 734 nm with a UV Mini 1240 Shimadzu spectrophotometer (Kyoto, Japan). The scavenging ability was expressed as EC_50_.

### 2.9. Sensory Evaluation of Bread

A 9-point hedonic scale was used to conduct the sensory analysis, where 1 indicated “extremely undesirable”, 5 represented “neither desirable nor undesirable”, and 9 indicated “extremely desirable”. The panel consisted of 36 trained individuals, including 25 women and 11 men (employees and students of SGGW in Warsaw), aged between 20 and 58 years. Participants were selected based on self-declarations of good health, no gluten allergies, and regular bread consumption. The bread samples were mechanically sliced into 1.2 mm thick pieces, coded, and served in random order to ensure an objective assessment. The following characteristics were evaluated: external appearance, color, texture, taste, aroma, and overall acceptability.

### 2.10. Statistical Analysis

The measurements were repeated a minimum of three times. Statistical analysis was performed using Statistica 13.3 (Palo Alto, CA, USA). An analysis of variance was conducted, and Tukey’s test was used to identify homogeneous groups, with a significance level set at α = 0.05.

## 3. Results and Discussion

### 3.1. Physical Characteristics of Dough

One of the fundamental tests used to assess the physical properties of dough is farinographic analysis, which allows for the determination of flour water absorption and key parameters related to dough development during mixing. These parameters are often linked to the baking quality of the flour and primarily depend on the content of gluten proteins [9] and the additives used in the flour [29]. The results of the analysis of both the control sample and the dough with CC (Table 1) showed that the addition of CC resulted in a decrease in water absorption (approximately 2% with a 10% addition), proportional to its content. However, it did not have a statistically significant influence on the development time of dough, which ranged from 1.6 to 1.8 min. The addition of powdered cereal ingredients with a high fiber content (above 50%) to wheat flour generally leads to an increase in its water absorption capacity [30]. CC contained about 20.0% fiber (dry matter), which suggests that other components of the coffee, such as proteins and carbohydrates, likely contributed to the reduction in the flour’s water absorption. Notably, the addition of CC significantly increased the dough stability parameter. A 2% addition resulted in more than a twofold increase in stability of dough related to the control sample (2.9 and 6.5 min, respectively). A higher addition of CC (4% and above) did not significantly affect dough stability, which remained at 8.1–8.3 min. Long dough development times and high dough stability typically indicate good flour quality for bread production [31]. In contrast, replacing wheat flour with 8% or 10% CC led to a significant increase in dough softening compared to the control sample, indicating a weakening of the dough structure due to the reduced gluten protein content caused by the introduction of fiber and gluten-free raw materials. Additionally, as Biernacka et al. [20] demonstrated, the components in CC hinder the formation of a strong gluten network. Similar trends were observed in studies by other authors [11,13,32,33], who investigated the properties of wheat dough with the addition of fiber-rich ingredients, such as powdered onion skin, freeze-dried fruit pomace, sumac, or tiger nut flour.

To assess the impact of the fermentation process on the extensibility and elasticity of dough, an extensograph analysis was performed. Long fermentation times allow for the observation of changes in the physical characteristics of the dough, which result from slow biochemical processes driven by both internal factors (such as the natural biochemical activity of the flour) and external factors (including baking improvers, nutrients, and health-promoting additives) [9]. Extensographic analysis of dough allows for the assessment of its elasticity and extensibility during stretching, which is crucial for determining its ability to form during processing. This analysis also provides information about the dough’s energy, its resistance to stretching, and its ability to return to its original shape, all of which are important for the quality of baked products. The results of the analysis of the control dough and the dough enriched with CC (Table 2) revealed that the addition of CC had no effect on the dough energy measured after 45 and 90 min of fermentation, but it significantly affected the value of this parameter after 135 min of fermentation. After 45 min of fermentation, the dough energy ranged from 110 cm^2^ (control sample) to 98 cm^2^ (with 10% coffee addition). After 90 min of fermentation, the dough energy slightly increased (99–118 cm^2^), whereas after 135 min, it decreased (82–112 cm^2^). After 135 min of fermentation, the dough energy with 8% and 10% CC addition was significantly lower compared to the doughs containing 2% and 4% of the additive, indicating a reduced ability of the dough to expand in volume, and consequently a weaker leavening by CO_2_ produced during fermentation. The studies conducted by Miś et al. [9] suggest that high-fiber additives can affect dough energy in various ways. Interestingly, in these studies, the addition of carob fiber increased dough energy, while the addition of whole grain oat flour reduced it. In our research, the enrichment of dough with CC had a significant impact on the values of the D coefficient, which is the ratio of resistance to extensibility of the dough. The values of this parameter for the control dough after 45, 90, and 135 min of fermentation ranged from 2.4 to 3.2. The addition of CC significantly increased the D coefficient, proportionally to its content. With the highest 10% addition, the D coefficient values were approximately three times higher than those of the control dough (ranging from 6.0 to 12.9). Regarding the baking quality of flour, it is assumed that a favorable ratio of resistance to extensibility (D coefficient) ranges from 3 to 5 [34]. In this case, the dough is elastic and extensible, showing appropriate mechanical strength. When the D value is below 3, the dough is less elastic, very fluid, and easily collapses even with minimal extension of the appropriate fermentation time. When the D coefficient is too high (above 5), the dough resists stretching, is less elastic, and the resulting bread has a dense, poorly porous crumb. Studies by Miś et al. [9] demonstrated the varied effects of high-fiber additives on the resistance of wheat dough to stretching and its extensibility. Dough with the addition of carob fiber exhibited greater resistance to stretching and was less extensible than the control sample (higher D values), while dough with the addition of whole grain oat flour was more extensible. The effect of CC in our study on the physical characteristics of dough was similar to the effect of carob fiber than that of whole grain oat flour.

In summary, the results of our study showed that the addition of coffee substitute to wheat flour had a significant influence on both farinograph and extensograph properties of the dough. It affected dough development time, stability, and softening. The increase in softening was particularly noticeable at higher levels of coffee substitute addition. At these higher levels, a decrease in dough energy was also observed, which is associated with a reduced ability of the dough to rise in volume. These changes can be explained by the lower amount of gluten in dough made from wheat flour and coffee substitute blends, compared to dough obtained from control dough. Additionally, the components of the coffee substitute, such as dietary fiber, hindered the formation of a strong gluten network.

### 3.2. Chemical Composition of Raw Materials and Bread

Cereal coffee, in comparison to wheat flour, exhibited slightly lower protein content (9.73% vs. 10.91% dry matter), lower levels of digestible carbohydrates (66.67% vs. 84.27% dry matter), approximately double the fat content (1.44% vs. 0.87% dry matter), three times higher ash content (1.86% vs. 0.64% dry matter), and six times higher total fiber content (20.30% vs. 3.31% dry matter) (Table 3). These changes in the chemical characteristics of wheat flour and CC influenced the chemical composition of the resulting breads. Specifically, the total fiber content in the bread increased, from 4.28% dry matter in the control bread to 6.65% dry matter when 10% of the wheat flour was replaced with CC. Both in the raw materials and in the breads produced from them, the content of insoluble fiber was higher. Dietary fiber, both soluble and insoluble, has a range of health benefits. Consuming adequate amounts of fiber reduces the risk of many lifestyle-related diseases and the likelihood of developing obesity [35]. Thus, CC may be a valuable health-promoting addition to bread. Differences in the content of other bread components enriched with CC were considerably smaller than those of fiber. In comparison with the control sample, with 10% of CC, the protein content decreased from 12.32% to 12.05% dry matter, the digestible carbohydrate content decreased from 81.85% to 79.10% dry matter, while the fat content increased from 0.71% to 1.15% dry matter, and the ash content increased from 0.84% to 1.05% dry matter.

### 3.3. Basic Properties of Bread

The results of the analysis of the basic bread properties are presented in Table 4. Baking losses decreased as the proportion of CC in the bread recipe increased (from 7.8% to 4.9%), which, in turn, led to an increase in bread yield (from 142.8% to 147.4%). A 2% addition of CC resulted in a significant reduction in baking losses and a marked increase in bread yield compared to the control sample. Similar trends were observed in previous studies where fiber-rich ingredients were used as additives in wheat bread recipes [9,13,36].

The specific volume, which refers to the bread volume per unit mass, is one of the key quality parameters. This parameter is associated with the texture and structure of the product. A higher specific volume usually corresponds to lower crumb density, with thinner cell walls. As a result, the bread has a softer and more delicate crumb, which increases consumer acceptance [37]. The specific volume of bread is also influenced by the added ingredients in the recipe [13,14,38]. When such ingredients are added to wheat flour, they typically weaken the gluten structure, leading to reduced loaf volume due to a decreased ability to retain gases, mainly CO_2_ and methane, in the crumb cells, which are released during dough fermentation [39]. A similar trend was observed with the addition of CC to wheat flour. The control bread exhibited the highest volume (369 cm^3^ per 100 g) and the lowest crumb density (0.24 g cm^−3^). As the proportion of CC in the bread formula increased, the volume of the bread decreased to 271 cm^3^ per 100 g with a 10% addition, and as a result, the density of crumb increased to 0.34 g cm^−3^.

### 3.4. Crumb Texture of Bread

Texture is one of the key factors in the assessment of bread quality. The hardness of the crumb increased with the rising proportion of CC in the bread (from 6.50 to 12.43 N), with a significant increase in hardness observed at a 4% addition of CC. The increase in crumb hardness can result from the higher content of dietary fiber and phenolic compounds. According to Pasrija et al. [40], these components may compete with starch for water, leading to greater crumb hardness. Other texture parameters, such as elasticity, springiness, and cohesiveness, decreased after the enrichment with CC, but these changes were less pronounced than the changes in hardness (Table 5). A significant decrease in elasticity and cohesiveness occurred with a 6% addition of CC, while a reduction in springiness was observed with a 10% addition of this ingredient.

### 3.5. Color of Raw Materials and Bread

**Figure 1:**
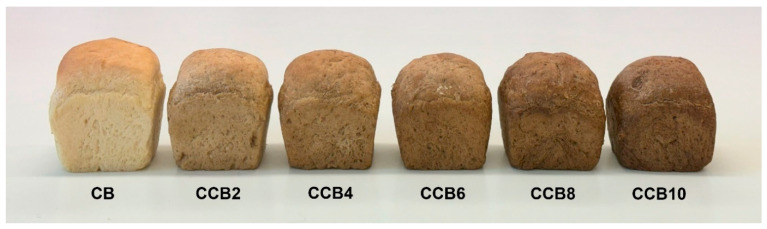
Appearance of bread loaves. CB—control bread, CCB2–CCB10—bread enriched with 2%, 4%, 6%, 8%, and 10% of cereal coffee.

**Figure 2:**
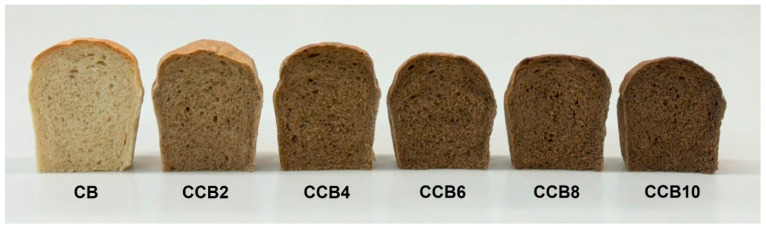
Appearance of the bread crumb. CB—control bread, CCB2–CCB10—bread enriched with 2%, 4%, 6%, 8%, and 10% of cereal coffee.

Color is one of the fundamental and most frequently assessed parameters in the qualitative evaluation of bread. The addition of CC resulted in darkening of both the crumb and crust, attributable to the significantly darker color of the coffee in relation to wheat flour (Table 6, Figure 1 and Figure 2). Even the lowest (2%) addition of CC caused a noticeable darkening of the crust color (a decrease in the L* parameter by approximately 5 units) and the crumb (a decrease in the L* parameter by approximately 15 units). Additionally, both the crumb and crust of the enriched product exhibited a higher proportion of red hues and a lower yellowness in relation to the control bread. These changes were directly related to the amount of coffee in the bread recipe. At the lowest coffee inclusion (2%), the total color difference (ΔE) between the enriched bread crust and the control sample was approximately 6 and increased with the amount of coffee in the formulation, reaching about 20 with a 10% substitution of wheat flour with CC. An even greater difference in ΔE was observed in the color of the crumb. At a 2% coffee inclusion, the ΔE between the enriched bread crumb and the control sample was around 16, and it increased with the addition level, reaching about 31 with a 10% substitution of wheat flour with CC. A ΔE value above 3 indicates that the color differences between the analyzed samples are noticeable to the average observer [32]. This suggests that even a small addition of CC to wheat flour significantly affects the color of both the crumb and crust.

The color of bread is influenced by both the raw materials used in its production and the production process [41,42]. In the case of CC, its color is determined by both the roasting process and the type of grain and additives used in its production. A similar effect of adding CC to wheat flour was observed in the production of pasta [20]. Other authors added green coffee powder to wheat flour and noted a slight effect of this addition on the brightness of the bread crumb. However, its yellowness increased, and the impact on the red hue was minimal [43].

### 3.6. Polyphenol Content and Antioxidant Activity of Raw Materials and Bread

Cereal coffee, compared to wheat flour, exhibited more than seventeen times higher content of phenolic compounds (17.46 vs. 1.00 mg GAE g DM^−1^) and significantly higher antioxidant activity against DPPH (EC_50_ of 14.43 vs. 183.93 mg DM mL^−1^) and ABTS (EC_5_0 of 31.87 vs. 223.80 mg DM mL^−1^) (Table 7). Refined wheat flour is poor in phenolic compounds. Enriching the bread recipe with CC resulted in an increase in the phenolic content of the bread, which was directly proportional to its content in the formulation. Even a 2% addition caused a significant increase in polyphenol content compared to the control sample (0.94 vs. 1.31 mg GAE g DM^−1^). At the maximum level of CC (10%) applied in the study, the increase in polyphenol content in the bread was nearly three times higher compared to the control sample (2.69 vs. 0.94 mg GAE g DM^−1^). The antioxidant capacity of the bread also increased with respect to both DPPH and ABTS. Research by Samsonowicz et al. [19] demonstrated that CC is particularly rich in gallic acid, a compound known for its potent anticancer, antioxidant, anti-inflammatory, and antimicrobial properties [44,45]. Additionally, gallic acid supports the cardiovascular system and is recommended for the prevention of chronic diseases [46]. CC also contains caffeic and ellagic acids, which are potent antioxidants that effectively remove reactive oxygen species and synthetic radicals, inhibit lipid oxidation, and chelate metal ions, as well as chlorogenic acid, which, among other effects, lowers blood sugar levels and exhibits antibacterial properties [16,18,19]. Similarly, other authors, when adding CC to pasta formulations, observed a significant increase in phenolic compound content in the enriched products, along with higher antioxidant capacity [20].

### 3.7. Sensory Evaluation Results

The results of the sensory evaluation of bread showed that the addition of CC, up to an inclusion level of 8%, did not have a significant impact on the assessed parameters of the bread, such as external appearance, texture, color, taste, and aroma (Table 8). Although this addition led to a reduction in flour water absorption and deterioration of the dough’s physical properties, producing loaves that were less risen, with a darker crust and crumb color (Figure 1 and Figure 2) and higher crumb hardness, it did not significantly affect the acceptability scores of the product. A significant decrease in the overall acceptability score, which dropped to 6.39, occurred only when wheat flour was replaced with 10% CC. For the control bread and the other samples, the acceptability scores ranged from 7.03 to 7.78. With the 10% addition of CC, the bread showed areas with a deformed crust and was the least well risen due to the weakening of the gluten structure caused by the addition of coffee, which contains over 20% fiber. Furthermore, bread with the highest content of CC received lower scores for taste, aroma, and texture in comparison to the other breads. Nevertheless, no notable differences in texture were observed between the different samples. As shown by other studies, the addition of fiber-rich raw materials to wheat flour generally results in lower scores for bread quality attributes, due to an increase in crumb hardness, decrease in loaf volume, and a deterioration in flavor and aroma qualities of the bread [47,48]. Therefore, for each additive, an optimal level should be established. As demonstrated in this study, the addition of CC to wheat bread should not exceed 8%.

## 4. Conclusions

Based on the conducted study, it was determined that CC significantly impacts the rheological properties of dough and the quality attributes of wheat bread. Increasing the proportion of cereal coffee in the formulation resulted in a reduction in the flour’s water absorption capacity and a weakening of the gluten structure, as evidenced by an increased degree of dough softening and a decrease in dough energy during stretching. These effects were particularly pronounced with 8% and 10% additions of CC. Moreover, the addition of CC diminished certain quality parameters of the bread, resulting in reduced volume and elasticity, alongside increased crumb firmness. However, the CC also enhanced bread yield and enriched it with fiber and bioactive compounds, leading to a notable increase in antioxidant activity and phenolic compound content. Additionally, the color of the crumb and crust became darker with an increase in red hues and a decrease in yellow hues as the concentration of CC increased. Importantly, despite the negative impact of CC on the physical properties of dough and bread volume, sensory analysis demonstrated a high level of acceptability for bread enriched with green coffee, with acceptability comparable to that of the control bread up to an 8% inclusion in wheat flour.

## Figures and Tables

**Table 1:** Water absorption and farinograph properties of dough.

Sample	Water Absorption [%]	Development Time [minutes]	Stability of Dough [minutes]	Degree of Softening [FU]
CD	55.0 ± 0.24 ^a^	1.8 ± 0.05 ^a^	2.9 ± 0.37 ^b^	69 ± 3.60 ^b^
CCD2	54.9 ± 0.25 ^a^	1.8 ± 0.09 ^a^	6.5 ± 1.66 ^a^	72 ± 0.94 ^ab^
CCD4	53.6 ± 0.21 ^b^	1.7 ± 0.17 ^a^	8.3 ± 0.42 ^a^	72 ± 4.99 ^ab^
CCD6	53.5 ± 0.25 ^b^	1.8 ± 0.14 ^a^	8.1 ± 0.46 ^a^	76 ± 2.62 ^ab^
CCD8	52.7 ± 0.00 ^c^	1.6 ± 0.09 ^a^	8.3 ± 0.57 ^a^	82 ± 3.09 ^a^
CCD10	52.2 ± 0.12 ^c^	1.6 ± 0.28 ^a^	8.1 ± 0.53 ^a^	82 ± 2.05 ^a^

CD—control dough, CCD2–CCD10—dough enriched with 2%, 4%, 6%, 8%, and 10% cereal coffee; the values marked with different letters ^a–c^ differ significantly (*p* < 0.05).

**Table 2:** Extensograph properties of dough.

Sample	Dough Energy (Area Under Curve) [cm^2^]			D [-]		
	45 min	90 min	135 min	45 min	90 min	135 min
CD	110 ± 3.0 ^a^	111 ± 7.0 ^a^	98 ± 2.0 ^abc^	2.4 ± 0.00 ^e^	3.0 ± 0.05 ^d^	3.2 ± 0.10 ^d^
CCD2	105 ± 11 ^a^	118 ± 6.5 ^a^	112 ± 5.5 ^a^	3.3 ± 0.10 ^d^	5.0 ± 0.15 ^c^	5.6 ± 0.45 ^cd^
CCD4	104 ± 7.5 ^a^	115 ± 1.5 ^a^	107 ± 3.0 ^ab^	4.4 ± 0.10 ^c^	7.3 ± 0.25 ^b^	7.4 ± 0.55 ^bc^
CCD6	99 ± 4.5 ^a^	101 ± 2.5 ^a^	97 ± 14.5 ^abc^	5.0 ± 0.10 ^b^	9.0 ± 0.10 ^ab^	8.9 ± 0.45 ^bc^
CCD8	100 ± 4.5 ^a^	99 ± 12 ^a^	82 ± 0.5 ^c^	5.2 ± 0.10 ^b^	8.7 ± 0.70 ^b^	9.7 ± 0.40 ^ab^
CCD10	98 ± 8.0 ^a^	100 ± 9.5 ^a^	85 ± 5.0 ^c^	6.0 ± 0.05 ^a^	10.8 ± 0.30 ^a^	12.9 ± 1.15 ^a^

CD—control dough, CCD2–CCD10—dough enriched with 2%, 4%, 6%, 8%, and 10% cereal coffee, D—dough coefficient; the values marked with different letters ^a–e^ differ significantly (*p* < 0.05).

**Table 3:** Chemical composition of the ingredients and the bread.

Sample	Moisture [%]	Protein [% DM]	Fat [% DM]	Ash [% DM]	Total Fiber [% DM]	Insoluble Fiber [% DM]	Soluble Fiber [% DM]	Available Carbohydrates [% DM]
WF	12.7 ± 0.0 ^A^	10.91 ± 0.03 ^A^	0.87 ± 0.01 ^B^	0.64 ± 0.02 ^B^	3.31 ± 0.08 ^B^	2.29 ± 0.07 ^B^	1.02 ± 0.01 ^B^	84.27 ± 0.13 ^A^
CC	7.1 ± 0.00 ^B^	9.73 ± 0.02 ^B^	1.44 ± 0.00 ^A^	1.86 ± 0.00 ^A^	20.30 ± 0.16 ^A^	14.10 ± 0.09 ^A^	6.20 ± 0.07 ^A^	66.67 ± 0.17 ^B^
CB	45.1 ± 0.09 ^a^	12.32 ± 0.00 ^a^	0.71 ± 0.01 ^d^	0.84 ± 0.00 ^c^	4.28 ± 0.06 ^d^	2.36 ± 0.09 ^c^	1.92 ± 0.04 ^c^	81.85 ± 0.07 ^a^
CCB2	44.9 ± 0.09 ^ab^	12.24 ± 0.0 ^ab^	0.86 ± 0.02 ^c^	0.93 ± 0.01 ^b^	4.83 ± 0.12 ^cd^	2.58 ± 0.00 ^bc^	2.25 ± 0.12 ^c^	81.14 ± 0.12 ^b^
CCB4	44.7 ± 0.05 ^b^	12.15 ± 0.02 ^bc^	0.90 ± 0.02 ^c^	0.94 ± 0.00 ^b^	4.89 ± 0.03 ^c^	2.88 ± 0.11 ^b^	2.03 ± 0.06 ^c^	81.12 ± 0.03 ^b^
CCB6	44.7 ± 0.08 ^b^	12.06 ± 0.01 ^c^	0.99 ± 0.02 ^b^	0.95 ± 0.01 ^b^	5.22 ± 0.17 ^c^	3.32 ± 0.08 ^a^	1.90 ± 0.09 ^bc^	80.78 ± 0.13 ^b^
CCB8	44.3 ± 0.05 ^c^	12.05 ± 0.01 ^c^	1.04 ± 0.01 ^b^	1.03 ± 0.01 ^a^	6.01 ± 0.04 ^b^	3.41 ± 0.02 ^a^	2.60 ± 0.02 ^b^	79.87 ± 0.04 ^c^
CCB10	44.2 ± 0.12 ^c^	12.05 ± 0.04 ^c^	1.15 ± 0.01 ^a^	1.05 ± 0.01 ^a^	6.65 ± 0.14 ^a^	3.59 ± 0.09 ^a^	3.01 ± 0.05 ^a^	79.10 ± 0.12 ^d^

WF—wheat flour, CC—CC, CB—control bread, CCB2–CCB10—bread enriched with 2%, 4%, 6%, 8%, and 10% of CC (cereal coffee); the values marked with different letters ^A,B^ or ^a–d^ differ significantly (*p* < 0.05).

**Table 4:** Basic properties of bread.

Sample	Baking Loss [%]	Bread Yield [%]	Specific Volume [cm^3^ 100^−1^ g]	Crumb Density [g cm^−3^]
CB	7.8 ± 0.05 ^a^	142.8 ± 0.74 ^c^	369 ± 3.74 ^a^	0.24 ± 0.01 ^d^
CCB2	7.2 ± 1.25 ^b^	144.4 ± 0.45 ^b^	341 ± 2.05 ^b^	0.26 ± 0.00 ^cd^
CCB4	7.1 ± 0.08 ^b^	144.5 ± 0.37 ^b^	329 ± 5.79 ^c^	0.28 ± 0.01 ^c^
CCB6	5.8 ±1.25 ^c^	144.6 ± 0.33 ^a^	299 ± 0.94 ^d^	0.31 ± 0.01 ^b^
CCB8	5.5 ± 0.16 ^d^	147.1 ± 0.78 ^a^	296 ± 0.81 ^d^	0.32 ± 0.01 ^b^
CCB10	4.9 ± 0.16 ^e^	147.4 ± 0.53 ^a^	271 ± 2.62 ^e^	0.34 ± 0.01 ^a^

CB—control bread, CCB2–CCB10—bread enriched with 2%, 4%, 6%, 8%, and 10% of cereal coffee; the values marked with different letters ^a–e^ differ significantly (*p* < 0.05).

**Table 5:** Crumb texture of bread.

Sample	Hardness [N]	Elasticity [-]	Springiness [-]	Cohesiveness [-]
CB	6.50 ± 0.33 ^d^	0.39 ± 0.0 ^a^	0.95 ± 0.00 ^a^	0.77 ± 0.0 ^a^
CCB2	6.98 ± 0.11 ^d^	0.38 ± 0.0 ^a^	0.94 ± 0.00 ^a^	0.76 ± 0.00 ^ab^
CCB4	7.67 ± 0.15 ^c^	0.37 ± 0.00 ^ab^	0.93 ± 0.00 ^ab^	0.75 ± 0.0 ^ab^
CCB6	10.04 ± 0.12 ^b^	0.35 ± 0.01 ^bc^	0.93 ± 0.01 ^ab^	0.71 ± 0.0 ^bc^
CCB8	10.20 ± 0.09 ^b^	0.35 ± 0.00 ^bc^	0.92 ± 0.00 ^ab^	0.69 ± 0.03 ^c^
CCB10	12.43 ± 0.29 ^a^	0.34 ± 0.0 ^c^	0.91 ± 0.02 ^b^	0.69 ± 0.02 ^c^

CB—control bread, CCB2–CCB10—bread enriched with 2%, 4%, 6%, 8%, and 10% of cereal coffee; the values marked with different letters ^a–d^ differ significantly (*p* < 0.05).

**Table 6:** Color coordinates of raw materials and bread.

Sample	L*	a*	b*	ΔE
WF	93.93 ± 0.13 ^A^	−0.37 ± 0.07 ^B^	14.16 ± 0.25 ^A^	-
CC	46.98 ± 0.79 ^B^	10.04 ± 0.21 ^A^	9.13 ± 0.93 ^B^	-
crust				
CB	64.50 ± 3.45 ^a^	7.15 ± 1.22 ^c^	22.51 ± 1.74 ^a^	-
CCB2	59.71 ± 2.63 ^b^	7.87 ± 0.35 ^c^	18.88 ± 0.94 ^b^	6.05
CCB4	52.75 ± 1.20 ^c^	8.18 ± 1.14 ^bc^	18.78 ± 1.17 ^b^	12.37
CCB6	51.27 ± 2.66 ^cd^	8.22 ± 0.86 ^bc^	16.36 ± 0.98 ^c^	14.63
CCB8	48.30 ± 1.33 ^de^	9.40 ± 0.42 ^ab^	15.04 ± 0.66 ^c^	17.98
CCB10	47.58 ± 1.30 ^e^	9.76 ± 0.44 ^a^	12.23 ± 0.77 ^d^	19.97
crumb				
CB	71.32 ± 1.76 ^a^	−0.25 ± 0.26 ^e^	14.99 ± 0.45 ^a^	-
CCB2	55.99 ± 1.44 ^b^	4.53 ± 0.30 ^d^	14.85 ± 0.51 ^a^	16.06
CCB4	49.88 ± 3.10 ^c^	6.25 ± 0.17 ^c^	14.67± 1.08 ^a^	22.41
CCB6	43.79 ± 2.97 ^d^	6.86 ± 0.19 ^b^	12.87 ± 1.10 ^b^	28.51
CCB8	42.62 ± 2.00 ^d^	7.14 ± 0.30 ^b^	12.73 ± 1.01 ^b^	29.72
CCB10	40.92 ± 1.73 ^d^	7.53 ± 0.10 ^a^	11.81 ± 0.62 ^b^	31.54

WF—wheat flour, CC—CC, CB—control bread, CCB2–CCB10—bread enriched with 2%, 4%, 6%, 8%, and 10% of cereal coffee; the values marked with different letters ^A,B^ or ^a–e^ differ significantly (*p* < 0.05).

**Table 7:** Antioxidant capacity of raw materials and bread.

Sample	TPC [mg GAE g DM^−1^]	EC_50 ABTS_ [mg DM mL^−1^]	EC_50 DPPH_ [mg DM mL^−1^]
WF	1.00 ± 0.04 ^B^	183.93 ± 0.21 ^A^	223.80 ± 3.61 ^A^
CC	17.46 ± 0.26 ^A^	14.43 ± 2.03 ^B^	31.87 ± 0.31 ^B^
CB	0.94 ± 0.04 ^f^	178.10 ± 1.25 ^a^	222.10 ± 3.50 ^a^
CCB2	1.31 ± 0.02 ^e^	98.77 ± 0.49 ^b^	166.37 ± 2.12 ^b^
CCB4	1.78 ± 0.04 ^d^	72.67 ± 1.10 ^c^	134.00 ± 1.35 ^c^
CCB6	2.09 ± 0.02 ^c^	54.10 ± 1.06 ^d^	123.87 ± 1.62 ^d^
CCB8	2.20 ± 0.02 ^b^	45.23 ± 0.66 ^e^	113.70 ± 1.56 ^e^
CCB10	2.69 ± 0.02 ^a^	37.60 ± 0.16 ^f^	97.20 ± 1.43 ^f^

WF—wheat flour, CC—CC, CB—control bread, CCB2–CCB10—bread enriched with 2%, 4%, 6%, 8%, and 10% of cereal coffee; the values marked with different letters ^A,B^ or ^a–f^ differ significantly (*p* < 0.05).

**Table 8:** Results of sensory evaluation of bread.

Sample	Appearance	Texture	Color	Taste and Aroma	Overall Acceptability
CB	8.17 ± 1.11 ^a^	7.46 ± 1.15 ^a^	7.67 ± 1.52 ^a^	7.21 ± 1.71 ^a^	7.63 ± 0.98 ^a^
CCB2	7.71 ± 0.98 ^ab^	7.46 ± 0.99 ^a^	8.21 ± 0.91 ^a^	7.75 ± 1.05 ^a^	7.78 ± 0.69 ^a^
CCB4	7.71 ± 0.93 ^ab^	7.71 ± 1.21 ^a^	8.13 ± 0.78 ^a^	7.75 ± 0.88 ^a^	7.83 ± 0.67 ^a^
CCB6	7.13 ± 1.20 ^bc^	7.33 ± 1.14 ^a^	7.46 ± 1.08 ^ab^	7.33 ± 1.25 ^a^	7.31 ± 0.83 ^a^
CCB8	7.13 ± 1.36 ^bc^	7.13 ± 1.20 ^a^	7.08 ± 1.44 ^ab^	6.79 ± 1.26 ^ab^	7.03 ± 0.93 ^ab^
CCB10	6.67 ± 1.52 ^c^	6.71 ± 1.49 ^a^	6.42 ± 1.91 ^b^	5.92 ± 1.87 ^b^	6.39 ± 1.31 ^b^

CB—control bread, CCB2–CCB10—bread enriched with 2%, 4%, 6%, 8%, and 10% of cereal coffee; the values marked with different letters ^a–c^ differ significantly (*p* < 0.05).

## Data Availability

The data presented in this study are available upon request from the first author (G.C.-P.). The data are not publicly available due to privacy restrictions.
